# Ambient fine particulate matter exposure influences oxidative stress and glucocorticoid concentrations in captive Asian elephants in Thailand

**DOI:** 10.1093/conphys/coag008

**Published:** 2026-02-17

**Authors:** Worapong Kosaruk, Janine L Brown, Chatchote Thitaram

**Affiliations:** Faculty of Veterinary Medicine, Chiang Mai University, 155 Moo 2, Mae Hia, Mueang Chiang Mai District, Chiang Mai 50100, Thailand; Elephant, Wildlife, and Companion Animals Research Group, Chiang Mai University, 239 Huay Kaew Road, Suthep, Muang Chiang Mai District, Chiang Mai 50100, Thailand; Elephant, Wildlife, and Companion Animals Research Group, Chiang Mai University, 239 Huay Kaew Road, Suthep, Muang Chiang Mai District, Chiang Mai 50100, Thailand; Centre for Species Survival, Smithsonian Conservation Biology Institute, 1500 Remount Road, Front Royal, VA 22630, USA; Faculty of Veterinary Medicine, Chiang Mai University, 155 Moo 2, Mae Hia, Mueang Chiang Mai District, Chiang Mai 50100, Thailand; Elephant, Wildlife, and Companion Animals Research Group, Chiang Mai University, 239 Huay Kaew Road, Suthep, Muang Chiang Mai District, Chiang Mai 50100, Thailand

**Keywords:** Asian elephant, faecal glucocorticoids, oxidative stress, PM_2.5_ exposure, pollution

## Abstract

Asian elephants, an iconic flagship species, are increasingly exposed to seasonal pollution and ambient fine particulate matter (PM_2.5_) due to land burning and regional air pollution across Northern Thailand. Unlike humans and domesticated animals, captive elephants often spend prolonged periods outdoors with minimal air quality or mitigation measures, yet the physiological consequences of chronic PM_2.5_ exposure remain poorly understood. This study investigated how daily PM_2.5_ levels affected oxidative stress and physiological stress biomarkers in Asian elephants involved in tourist activities in Thailand. A total of 27 elephants from seven tourist facilities in Northern Thailand were repeatedly sampled for serum 8-hydroxy-deoxyguanosine (8-OHdG, a marker of oxidative DNA damage), serum malondialdehyde (MDA, a marker of lipid peroxidation) and faecal glucocorticoid metabolites (fGCM, a marker of stress). Daily PM_2.5_ concentrations were classified into tertiles (low, moderate, high). Linear mixed-effects models were used to test associations between PM_2.5_ and each biomarker, with elephant ID and camp as random intercepts. Elephants exposed to high PM_2.5_ showed approximately 40% higher DNA damage and 35% higher stress hormone concentrations compared to low PM_2.5_ conditions. In contrast, lipid peroxidation concentrations were about 15% lower under high PM_2.5_ conditions, suggesting possible compensatory antioxidant responses. The strongest changes occurred when pollution increased from low to moderate levels, further increases produced smaller effects. These findings suggest that seasonal air pollution elevates stress hormones and triggers complex, at times counterintuitive, changes in oxidative biomarkers, likely due to physiological buffering in elephants, with potential health implications. Integrated multi-biomarker panels are therefore essential for accurately monitoring air quality impacts on captive megafauna. Proactive management should prioritize reducing exposure and providing nutritional support during peak pollution conditions to mitigate cumulative stress.

## Abbreviations

AIC, Akaike Information Criterion
CI, Confidence interval
fGCM, Faecal glucocorticoid metabolites
HPA axis, Hypothalamic–pituitary–adrenal axis
LMM, Linear mixed-effects model
MDA, Malondialdehyde
PM2.5, Particulate matter less than 2.5 micrometres in diameter
ROS, Reactive oxygen species
SE, Standard error
WAQI, World Air Quality Index
WHO, World Health Organization
8-OHdG, 8-hydroxy-deoxyguanosine

## Introduction

Fine particulate matter (PM_2.5_) pollution is an escalating concern for both human and animal health, especially in Southeast Asia, where seasonal biomass burning, urban emissions and stagnant weather conditions converge to generate prolonged pollution episodes (Amnuaylojaroen et al., 2022; Sirithian and Thanatrakolsri, 2022). PM_2.5_, airborne particles smaller than 2.5 micrometres, can penetrate deeply into the respiratory tract, inducing systemic physiological effects primarily through the generation of reactive oxygen species (ROS) (Amnuaylojaroen and Parasin, 2024). In mammals, inhaled particulates stimulate ROS production that can damage nucleic acids and cellular membranes (Rao et al., 2018; Amnuaylojaroen and Parasin, 2024). Among oxidative stress biomarkers, 8-hydroxy-deoxyguanosine (8-OHdG) is a widely recognized indicator of oxidative DNA damage in humans and wildlife, including Asian elephants (Kosaruk et al., 2023a). Likewise, malondialdehyde (MDA) is commonly used as a marker of lipid peroxidation, reflecting ROS-driven membrane damage (Pratiwi and Haryanto, 2020; Kosaruk et al., 2023b). Elevated 8-OHdG and MDA have been linked to adverse health outcomes such as impaired immune competence and heightened disease susceptibility in diverse mammalian species exposed to pollution and urban pollutants (Erb et al., 2018; Pan et al., 2021; Robertson et al., 2023; Sabir et al., 2025).

Beyond oxidative pathways, air pollution can also stimulate the hypothalamic–pituitary–adrenal (HPA) axis, elevating circulating glucocorticoids as a systemic stress response (Thomson, 2019; Miller et al., 2020). In large mammals, faecal glucocorticoid metabolites (fGCMs) are a validated, non-invasive proxy for adrenal activity and have been widely adopted to monitor stress in both free-ranging and intensively managed wildlife (Kosaruk et al., 2020, 2023b; Karaer et al., 2023). While numerous human and laboratory mammal studies demonstrate that PM_2.5_ exposure elevates both oxidative damage and glucocorticoid output (Rao et al., 2018; Gangwar et al., 2020; Liu et al., 2022; Kim et al., 2024; Sabir et al., 2025), studies are limited in free-ranging wildlife. Meta-analyses in humans have shown robust associations between PM_2.5_ and markers of oxidative stress and cortisol elevation, reinforcing the physiological burden of particulate exposure (Lobato et al., 2024; Wathanavasin et al., 2024; Hamzah et al., 2025). However, comparable data for non-human species, particularly long-lived terrestrial megafauna such as elephants, remain extremely scarce. Understanding these effects in wildlife is especially urgent for large, long-lived mammals frequently exposed to human activities. For instance, urban coyotes have been shown to exhibit elevated hair cortisol concentrations in polluted cityscapes (Robertson et al., 2023).

Asian elephants (*Elephas maximus*) are particularly vulnerable to chronic and acute anthropogenic stressors because of their large body size, high absolute metabolic demands and constant proximity to intensive human activities such as tourism and conservation settings (Norkaew et al., 2018, 2019). In Northern Thailand, captive elephants are typically housed outdoors year-round, with no indoor enclosures or effective air filtration systems. This means that during high-pollution periods, elephants remain fully exposed to ambient PM_2.5_ without any practical barriers or protective interventions. Unlike humans, who may retreat indoors or wear masks, these animals have no means to avoid direct inhalation of particulates. Such constant exposure may increase the likelihood of cumulative respiratory irritation, systemic oxidative imbalance and chronic stress activation. Yet, the short- and medium-term physiological consequences of repeated pollution exposure in elephants remain poorly quantified. To date, there has been no systematic attempt to measure how ambient fine particulates influence oxidative pathways or adrenal hormone activity under realistic management conditions. This knowledge gap limits the capacity of veterinarians, elephant camp managers and policy stakeholders to assess health risks and implement evidence-based management strategies that could mitigate pollution impacts on this flagship species.

To address this gap, the present study investigated the effects of ambient PM_2.5_ exposure on serum 8-OHdG, MDA and fGCM concentrations in captive Asian elephants managed across multiple tourist facilities in Chiang Mai province, Northern Thailand. By combining repeated, seasonally distributed sampling, validated oxidative and endocrine markers, and mixed-effects statistical models, we tested the hypothesis that increasing PM_2.5_ would lead to elevated oxidative damage and stress hormone biomarker levels. These findings provide critical baseline evidence for integrating air quality risks into practical elephant management and reinforce that pollution episodes pose a tangible but overlooked threat to the long-term health and welfare of captive elephants in Southeast Asia.

## Materials and Methods

### Study design and population

All procedures adhered to institutional animal welfare guidelines and were approved by the Animal Use Committee, the Faculty of Veterinary Medicine, Chiang Mai University (FVM, CMU) (Approval No. S7/2564).

**Figure 1:**
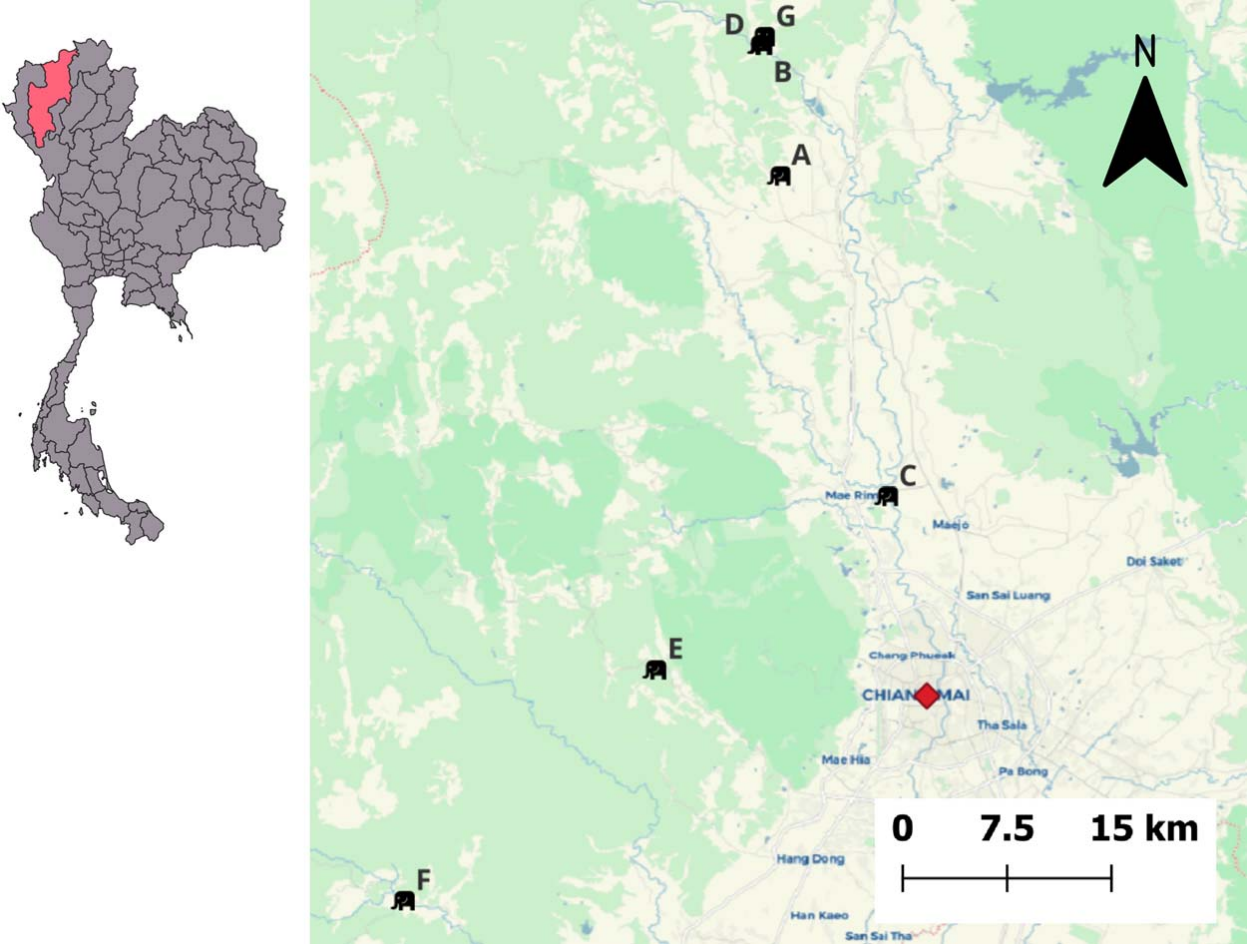
Study location showing the elephant camps (A to G) and PM_2.5_ monitoring station (red diamond) in this study (Chiang Mai, Thailand)

Twenty-seven captive Asian elephants housed across seven tourist facilities (or ‘elephant camps’) in Chiang Mai province, Thailand, participated in this study. All camps were situated in rural areas within 55 km of Chiang Mai city. Camps varied in size, proximity to PM monitoring stations and primary tourist activities (Table 1). A map of camps and the PM_2.5_ detector location is illustrated in Fig. 1. Elephants ranged from 2 to 60 years of age (mean ± SE: 14.3 ± 2.8 years), and included seven males (6.3 ± 1.24, range 3–11 years) and 20 females (17.2 ± 3.6, range 2–60 years). All elephants were healthy; no clinical signs were detected by experienced veterinarians during the sample collection period. The seven participating camps were selected based on existing research collaborations and availability of repeated health monitoring records, rather than geographic proximity to air monitoring infrastructure.

**Table 1:** General information of each elephant camp in the study

**Camp**	**Total elephants in each camp**	**Number of participating elephants (%)**	**Euclidean distance from PM station (km)**	**Main tourist activities**	**Total samples (serum/faecal)**	**Mean samples per elephant (serum/faecal)**
A	43	9 (33.3%)	37.64	Observation, feeding, mud playing, bathing	73/73	8.1/8.1
B	32	5 (18.5%)	46.83	Riding with saddle	30/35	6/7
C	6	1 (3.7%)	14.05	Observation and feeding	5/4	5/4
D	52	4 (14.8%)	46.79	Riding with saddle	48/44	12/11
E	38	2 (7.4%)	18.26	Observation, feeding, forest trekking, bathing	24/24	12/12
F	30	5 (18.5%)	37.83	Riding with saddle, elephant show	59/57	11.8/11.4
G	15	1 (3.7%)	47.29	Riding without saddle, forest trekking, bathing	12/12	12/12

The primary diet consisted of fresh roughage (e.g. grass and corn stalks), occasionally supplemented with fruits such as bananas and sugar cane. All elephants participated in tourist activities, depending on the camp, such as observation-only, riding, feeding and bathing. All elephants were housed outdoors for 24 hours; no additional management provisions (e.g. water sprays) were provided.

### Sample collection and biomarker analyses

Each elephant was sampled (blood and faeces) monthly from July 2021 to September 2022 in the morning (0900 to 1100 hours). Blood samples were collected via standard venipuncture from an ear vein under manual restraint using a 21-G butterfly needle attached to a 10-mL syringe and transferred to red-top serum tubes for assessment of oxidative damage markers (8-OHdG and MDA). Faecal samples were collected opportunistically after spontaneous defecation and were typically retrieved from the floor within approximately 30 minutes of deposition. The topmost faecal bolus that had not contacted the ground, urine or other contaminations was selected to minimize environmental contamination. The entire bolus was mixed thoroughly, and 20 g subsamples were transferred into labelled zip-lock bags. All samples were placed into a cooled insulated box containing ice packs (estimated temperature 2–8°C) and transported (<2 hours) to the laboratory at FVM, CMU. Blood was centrifuged at 1500×g for 10 minutes to obtain serum. Serum and faecal samples were stored at −20°C until extraction and analysis. Across all camps, the estimated time from defecation to freezing was approximately 3–4 hours.

All biomarker analyses were conducted using assays validated for Asian elephants (Kosaruk et al., 2023a). Concentrations of 8-OHdG were assessed using commercial enzyme immunoassay kits (Cat #K059-H5, Arbor Assays, Michigan, United States), and MDA was measured using a thiobarbituric acid reactive substances (TBARSs) assay. Faecal samples were ethanol extracted and analyzed using a double-antibody corticosterone enzyme immunoassay that relied on an anti-corticosterone antibody (R006, Coralie Munro, Davis, CA) and corticosterone-HRP tracer (SCBI, Front Royal, VA).

### Ambient PM_2.5_ data

Daily PM_2.5_ concentrations were obtained from the World Air Quality Index (WAQI) project using one PM_2.5_ monitoring station at Chiang Mai city (https://aqicn.org/city/chiang-mai/; accessed on June 2025), which was within 50 km of the elephant camp locations. The location of elephant camps and the CMU monitoring station are displayed in Fig. 1. Straight-line (Euclidean) distance from each elephant camp to the PM_2.5_ monitoring station was calculated in QGIS using WGS84 coordinates (Table 1).

To classify PM_2.5_ exposure levels, daily PM_2.5_ concentrations were grouped into tertiles using the 33rd and 66th percentiles of the sample distribution (low PM_2.5_: ≤14.48 μg/m^3^, moderate PM_2.5_: >14.48 to ≤22.56 μg/m^3^, high PM_2.5_: >22.56 μg/m^3^) to enable detection of dose–response relationships within the sample’s observed range. A secondary classification using the WHO 24-hour mean guideline of 35 μg/m^3^ was tested for sensitivity, showing consistent but less stable estimates due to imbalanced sample size (data not shown).

### Statistical analysis

Descriptive statistics were calculated and presented as mean ± standard error (SE). Linear mixed-effects models (LMMs) were fitted to examine associations between PM_2.5_ exposure and each biomarker outcome. Model selection was guided by Akaike’s Information Criterion (AIC). To account for possible time lags between exposure and biomarker responses, daily PM_2.5_ concentrations were shifted by 0–3 days (Lag 0 = same day of sample collection, Lag 1 = 1 day prior, Lag 2 = 2 days prior, Lag 3 = 3 days prior). This lag structure was selected based on expected biomarker kinetics (e.g. gut transit time for fGCM and serum oxidative responses for 8-OHdG and MDA) and to balance model parsimony with explanatory relevance (Wheeler et al., 2013; Greene et al., 2019; Li et al., 2020; Di Francesco et al., 2021; Kim et al., 2024). The best lag for each biomarker was chosen based on the lowest AIC (8-OHdG: Lag 0; MDA: Lag 3; fGCM: Lag 2) (Supplementary Table S1). The final models included the selected lag term, PM_2.5_ tertile category and their interaction as fixed effects, with age class and sex as covariates. Elephant ID and camp were included as random intercepts to account for repeated measures and site-level clustering.

Model residuals were visually inspected using Q–Q plots and residual-vs-fitted plots to assess normality and homoscedasticity (Supplementary File 1). No major deviations from normality were observed. Temporal autocorrelation in residuals was examined using autocorrelation function (ACF) plots, which showed no substantial autocorrelation (Supplementary File 1). Additional model fit was assessed using marginal and conditional R^2^ values computed with the `performance` package (v0.15.2) in R, quantifying variance explained by fixed effects alone and in combination with random effects (Supplementary Table S2). Predictor multi-collinearity was examined using generalized variance inflation factors (GVIF) from the car package (v3.1.3); all GVIF^1/2df^ were below 1.2, indicating acceptable independence among predictors. Effect sizes were reported with corresponding 95% confidence intervals. All analyses were performed in R (v4.5.1) using the lme4 (v1.1.37), lmerTest (v3.1.3) and related packages. To visualize the raw relationships between PM_2.5_ and each biomarker, scatterplots using continuous PM_2.5_ values across all lag periods (Lag 0–3) were included in Supplementary File 2.

## Results

### Descriptive statistics

**Figure 2:**
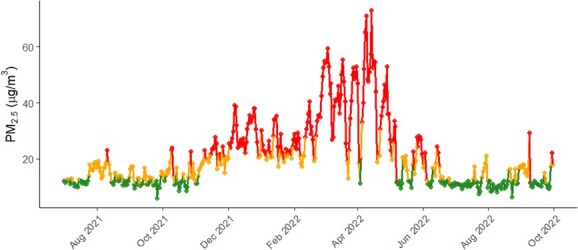
Daily mean PM_2.5_ concentrations measured during the study period (July 2021–September 2022). Lines and dots are colour-coded by pollution category based on tertiles: low PM_2.5_ (green), moderate PM_2.5_ (yellow) and high PM_2.5_ (red)

**Figure 3:**
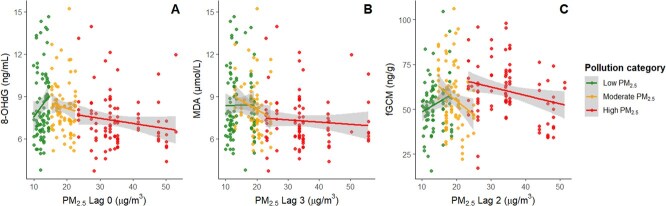
Associations between daily PM_2.5_ exposure and three stress biomarkers across pollution categories. The relationship between daily PM_2.5_ and each biomarker is shown at the lag period with the strongest association: (**A**) faecal 8-OHdG with PM_2.5_ Lag 0, (**B**) MDA with PM_2.5_ Lag 3 and (**C**) fGCM with PM_2.5_ Lag 2. Each point represents an individual observation, coloured by PM_2.5_ tertile category (low PM_2.5_ = green, moderate PM_2.5_ = yellow, high PM_2.5_ = red). Linear trend lines and 95% confidence intervals (shaded area) are shown for each category. 8-OHdG; 8-hydroxy-deoxyguanosine, MDA; malondialdehyde, fGCM; faecal glucocorticoid metabolites

A total of 263 serum and 252 faecal samples were collected from 27 elephants. On average, 9.3 serum and 9.2 faecal samples were collected per elephant (range: 4–12 for both matrices). Daily mean PM_2.5_ concentrations across the study period are displayed in Fig. 2. Concentrations averaged 22.08 ± 10.77 μg/m^3^ and ranged from 9.84 to 52.96 μg/m^3^ during the study period. Average PM_2.5_ concentrations were 12.50 ± 1.39 μg/m^3^ (range, 9.84 to 14.48 μg/m^3^) for low PM_2.5_, 18.76 ± 2.43 μg/m^3^ (range, 15.43 to 22.56 μg/m^3^) for moderate PM_2.5_, and 35.05 ± 8.03 μg/m^3^ (range, 23.51 to 52.96 μg/m^3^) for high PM_2.5_ concentrations. Mean biomarker concentrations categorized by PM_2.5_ category are presented in Table 2 and Fig. 3.

**Table 2:** Mean ± standard error, and range of 8-OHdG, MDA and fGCM concentrations categorized by pollution category^a^

**Pollution category**	**8-OHdG (ng/mL)**		**MDA (μmol/L)**		**fGCM (ng/g)**	
	**Mean ± SE**	**Range**	**Mean ± SE**	**Range**	**Mean ± SE**	**Range**
Low PM_2.5_ (*N* = 90)	8.38 ± 0.26	3.79–14.66	1.93 ± 0.05	1.34–4.18	52.53 ± 1.55	15.80–104.36
Moderate PM_2.5_ (*N* = 84)	8.24 ± 0.20	5.14–15.23	1.69 ± 0.04	1.04–2.99	56.39 ± 1.89	22.16–106.08
High PM_2.5_ (*N* = 88)	7.27 ± 0.20	3.73–13.96	1.72 ± 0.04	1.04–2.99	60.21 ± 1.70	17.24–97.87
Total	7.94 ± 0.07	3.73–15.23	1.78 ± 0.01	1.04–4.18	56.49 ± 0.54	15.80–106.08

Abbreviations: 8-OHdG; 8-hydroxy-deoxyguanosine, MDA; malondialdehyde, fGCM; faecal glucocorticoid metabolites

a These data represent unadjusted descriptive values by PM_2.5_ tertiles and are not stratified by lag model structure.

**Table 3:** Linear mixed-effects model results showing fixed effect estimates (β), standard errors (SE), *t*-values, degree of freedom (df), 95% coefficient interval (CI) and *P*-values for the effects of PM_2.5_ exposure on stress biomarkers

**Predictor**	**Estimate (β)**	**SE**	***t*-value**	**df**	**95% CI**	***P*-value**
8-OHdG (lag 0)						
Intercept	6.469	1.653	3.914	142.738	(2.90, 8.78)	<0.01^**^
Age	−0.155	0.093	−1.668	20.508	(−0.09, 0.01)	0.11
Sex						
Female	(Reference)					
Male	−0.398	0.859	−0.463	20.12	(−1.96, 1.47)	0.648
PM_2.5_	0.251	0.107	2.338	225.25	(0.04, 0.463)	0.02^*^
Pollution category						
Low	(Reference)					
Moderate	3.821	1.801	2.122	224.528	(0.30, 7.39)	0.035^*^
High	3.516	1.491	2.358	224.945	(0.59, 6.46)	0.019^*^
PM_2.5_ × Pollution category						
PM_2.5_ × Low	(Reference)					
PM_2.5_ × Moderate	−0.293	0.124	−2.366	224.512	(−0.54, −0.05)	0.019^*^
PM_2.5_ × High	−0.287	0.109	−2.635	225.317	(−0.50, −0.07)	0.009^**^
MDA (lag 3)						
Intercept	2.187	0.201	10.878	103.276	(1.82, 2.56)	<0.01^**^
Age	−0.012	0.012	−1.047	14.207	(−0.01, 0.0003)	0.313
Sex	−0.092	0.107	−0.856	12.726		0.408
Female	(Reference)					
Male					(−0.33, 0.09)	
PM_2.5_	−0.009	0.012	−0.733	228.054	(−0.03, 0.01)	0.464
Pollution category						
Low	(Reference)					
Moderate	0.388	0.3	1.293	221.199	(−0.20, 0.98)	0.197
High	−0.717	0.22	−3.263	224.172	(−1.14, −0.27)	0.001^**^
PM_2.5_ × Pollution category						
PM_2.5_ × Low	(Reference)					
PM_2.5_ × Moderate	−0.032	0.018	−1.793	220.879	(−0.07, 0.003)	0.074
PM_2.5_ × High	0.019	0.013	1.539	227.408	(−0.006, 0.04)	0.125
fGCM (lag 2)						
Intercept	38.779	9.807	3.954	198.694	(19.7, 57.6)	<0.01^**^
Age	−0.202	0.379	−0.534	26.878	(−0.30, 0.13)	0.597
Sex						
Female	(Reference)					
Male	1.597	3.285	0.486	21.125	(−5.50, 7.72)	0.632
PM_2.5_	1.079	0.681	1.584	230.732	(−0.25, 2.42)	0.115

(Continued)

**Table 3:** Continued

**Predictor**	**Estimate (β)**	**SE**	***t*-value**	**df**	**95% CI**	***P*-value**
Pollution category						
Low	(Reference)					
Moderate	39.598	14.688	2.696	224.786	(10.80, 68.70)	0.008^**^
High	38.534	11.482	3.356	229.039	(16.10, 61.20)	0.001^**^
PM_2.5_ × Pollution category						
PM_2.5_ × Low	(Reference)					
PM_2.5_ × Moderate	−2.27	0.893	−2.541	225.037	(−4.04, −0.52)	0.012^*^
PM_2.5_ × High	−1.566	0.707	−2.216	230.552	(−2.96, −0.18)	0.028^*^

Abbreviations: 8-OHdG; 8-hydroxy-deoxyguanosine, MDA; malondialdehyde, fGCM; faecal glucocorticoid metabolites

### 8-OHdG

DNA damage levels increased significantly with higher PM_2.5_ concentrations at Lag 0 (AIC = 943.02) (Supplementary Table S1). The LMM results of 8-OHdG concentrations are detailed in Table 3. Age did not show a clear effect on 8-OHdG concentrations, and no sex difference was detected. However, increased daily PM_2.5_ (Lag 0) was significantly associated with higher DNA damage, with an estimated increase of ~0.25 ng/mL per unit increase (*P* < 0.05). Animals sampled under moderate and high PM_2.5_ conditions exhibited approximately 40% greater DNA damage compared to those sampled under low PM_2.5_ conditions. The interaction term indicated that when background pollution was already high, the incremental effect of additional PM_2.5_ was reduced by about 30% per unit increase (see Fig. 3A for the dose–response trend).

### MDA

Model fit was strongest at Lag 3 for MDA (AIC = 269.70) (Supplementary Table S1). The LMM results of MDA concentrations are shown in Table 3. Age and sex were non-significant predictors. Daily PM_2.5_ alone showed no direct linear effect (Lag 3). However, MDA levels under high PM_2.5_ were ~ 15% lower than those under low PM_2.5_, indicating a possible dampening effect of chronic pollution exposure. The PM × pollution interaction showed a weak trend but did not meet conventional significance thresholds (*P* ≈ 0.07). Patterns are illustrated in Fig. 3B.

### Faecal glucocorticoid metabolites

For faecal glucocorticoid metabolites (fGCM), Lag 2 provided the lowest AIC (AIC = 2062.21) (Supplementary Table S1). The LMM results of fGCM concentrations are shown in Table 3. No clear age or sex effect was observed for fGCM. PM_2.5_ (Lag 2) alone was not significantly related to fGCM concentrations. However, average fGCM levels were ~ 35% higher under moderate PM_2.5_ and high PM_2.5_ compared to low PM_2.5_. The negative interaction term indicated that additional daily PM_2.5_ contributed less to the stress hormone response during sustained pollution periods than under clearer conditions. Full patterns appear in Fig. 3C.

## Discussion

This study provides the first evidence supporting that acute exposure to ambient PM_2.5_ pollution significantly impacts oxidative and physiological stress of captive elephants in Thailand. Our linear mixed-effects models revealed complex and biomarker-specific responses to pollution-related PM_2.5_ exposure in elephants. Serum 8-OHdG initially increased slightly with rising PM_2.5_ under low-pollution conditions, but showed a more pronounced decline under moderate- and high-pollution conditions due to significant negative interactions. MDA concentrations declined consistently as PM_2.5_ increased, reaching the lowest levels under high-pollution conditions. Conversely, fGCM concentrations were consistently elevated during periods of higher PM_2.5_ (i.e. under high-pollution conditions). These results confirm and extend previous findings from captive mammal and human studies, emphasizing PM_2.5_ as a critical environmental stressor capable of compromising captive wildlife health (Chen et al., 2020; Liu et al., 2022;Kim et al., 2024; Sabir et al., 2025).

A non-linear pattern was observed for serum 8-OHdG at low PM_2.5_ concentrations, which then declined at higher pollution levels. The initial positive association between PM_2.5_ and 8-OHdG is consistent with the well-established idea that air pollutants induce oxidative DNA damage, as air particulate matter stimulates the production of hydroxyl radicals and other ROS upon lung deposition, contributing to systemic absorption (Kim et al., 2024; Sabir et al., 2025). Indeed, controlled human and animal studies show that short-term increases in fine particulates are linked to higher 8-OHdG in blood and urine, reflecting oxidative stress on DNA (Chen et al., 2020; Liu et al., 2022; Zhang et al., 2022; Atikah et al., 2024). In our study, elephants under low-pollution conditions (relatively clean air) showed an expected small increase in 8-OHdG as PM_2.5_ rose. However, as pollution worsened, the relationship inverted: at moderate and high PM_2.5_ levels, 8-OHdG concentrations actually fell. This reversal suggests that elephants may activate compensatory antioxidant mechanisms under severe pollution conditions. Such non-linear responses have analogues in other studies. For example, in human clinical contexts (e.g. hypertensive patients), initial increases in oxidative stress markers with pollution or stress have been followed by apparent decreases, interpreted as induction of antioxidant defences or changes in repair pathways (Christiani, 2009; Rao et al., 2018). Elephants possess exceptionally robust antioxidant systems, maintaining high serum albumin levels and elevated activities of enzymes such as glutathione peroxidase (GPx) and catalase, which effectively neutralize ROS (Kosaruk et al., 2023a). Serum albumin itself is a major circulating antioxidant (Belinskaia et al., 2021; Watanabe et al., 2025), and previous studies have noted that elephants often have abundant circulating antioxidants (Kosaruk et al., 2023a, 2023b). We hypothesize that when pollution becomes severe, elephants upregulate these defences so effectively that net circulating DNA damage (8-OHdG) decreases. In effect, initial oxidative insults may trigger a physiological “over-compensation,” resulting in lower detectable DNA damage at high PM_2.5_ despite continued exposure. This interpretation aligns with the notion that chronic or intense pollution can induce adaptive responses (e.g. increased repair or scavenging) that mask direct pollutant effects (Gangwar et al., 2020; Niu et al., 2020). It is important to emphasize, however, that this hypothesis does not dismiss the observed negative associations as biologically irrelevant. Rather, it highlights that lower circulating damage markers under high pollution should be interpreted cautiously and may signal compensatory processes rather than the absence of pollution effects. Future studies, thus, are warrant to further assess oxidant/antioxidant markers to confirm this.

Similar to 8-OHdG, serum MDA declined as PM_2.5_ increased, particularly under the heaviest pollution conditions. This was also contrary to the expectation since humans and animal studies typically report that MDA increases with pollution exposure (Hu et al., 2017; He et al., 2020). For example, a meta-analysis in humans found that each 10 μg/m^3^ short-term rise in PM_2.5_ was associated with a significant 1.6% increase in blood MDA (Li et al., 2020), reflecting greater lipid oxidative damage. A recent study in Chiang Mai (the same location as our study) also found urinary MDA concentrations were significantly higher during high-PM_2.5_ than low-PM_2.5_ periods (Sabir et al., 2025). Thus, declining MDA under heavy pollution contradicts this expectation. By contrast, our results echo a few reports of negative associations. In Jakarta transport workers (Pratiwi and Haryanto, 2020), PM_2.5_ exposure was negatively associated with urinary MDA. Authors noted the contrary findings to other studies, which may have been confounded by body mass index; overweight/obese drivers were at increased risk for oxidative stress in environments with high particulate pollution (Pratiwi and Haryanto, 2020). Whether similar factors play a role in elephants is unclear and needs to be investigated further. Notably, seasonal patterns in elephants show higher MDA during hot, dry seasons (when pollution is often worse) (Kosaruk et al., 2023a), reinforcing that under normal seasonal variation, elephants produce more lipid peroxidation when heat and pollution co-occur.

Our paradoxical MDA decline may also reflect behavioural or physiological adjustments. Elephant daily activities are controlled by mahouts and related to tourist numbers. During intense pollution, tourist numbers are often lower (Evrard and Mostafanezhad, 2023; Nonthapot et al., 2024), and consequently, elephants may work less. Such indirect reductions in activity could lead to less free-radical-induced lipid damage, as suggested in other reviews (Hahad et al., 2021; Relić and Đukić-Stojčić, 2023). Alternatively, chronic pollution could upregulate lipid-antioxidant defences (such as superoxide dismutase [SOD], catalase and GPx) more effectively than in short-term exposures (Rao et al., 2018; Cipryan et al., 2024). Increased clearance or excretion of peroxidation products might also occur. Another factor is timing: the strongest PM_2.5_ effects on MDA might occur soon after smoke onset (Li et al., 2020), but our samples could reflect a lag as pollution persists. Indeed, our analysis indicated that MDA responded with a delay (peaking at Lag 3), suggesting that lipid peroxidation spiked early and then was cleared by the time of measurement. Finally, systemic glucocorticoid changes could influence lipid metabolism: high cortisol can shift the body away from lipid oxidation pathways (Costantini et al., 2012; Garre-Morata et al., 2024). In sum, while human studies predict rising MDA with PM_2.5_, our results show that elephants under sustained pollution ultimately exhibit lower measured MDA, likely due to rapid compensatory or metabolic changes that limit ongoing lipid damage.

In contrast to oxidative markers, fGCM showed a clear positive relationship with PM_2.5_ levels, increasing significantly as pollution intensified, with elephants under high-pollution conditions exhibiting markedly higher stress hormone concentrations. This pattern aligns with the idea that poor air quality acts as a physiological stressor. Fine particulates are known to activate the hypothalamic–pituitary–adrenal (HPA) axis in mammals; human trials consistently report that increased PM_2.5_ exposure triggers increases in cortisol and related hormones (Li et al., 2017; Thomson, 2019; Cowell et al., 2023). In one randomized crossover study, participants breathing higher PM_2.5_ air for days showed significant increases in cortisol, cortisone and catecholamines (Li et al., 2017). Likewise, animal studies have demonstrated that airborne pollutants can induce endocrine stress responses through the HPA axis (Hu et al., 2021; Pan et al., 2021; Liu et al., 2024).

The surge in stress hormones under heavy pollution is ecologically and welfare-relevant. Chronic elevations in glucocorticoids can have detrimental effects on immune function, reproduction and overall health. In wildlife and livestock, sustained high cortisol correlates with susceptibility to disease, reduced fertility and metabolic problems (Herman et al., 2016; Thomson, 2019). Thus, pollution-induced fGCM elevation signals a potentially serious physiological burden. It also highlights the importance of considering not only oxidative damage but also endocrine stress when assessing the effects of air pollution on animals.

Several unmeasured environmental and management factors may have also influenced stress and oxidative biomarkers. These include the intensity of tourist activities, social group structure and enclosure/camp characteristics (Brown et al., 2019; Norkaew et al., 2019; Kosaruk et al., 2020). Because those variables could not be consistently quantified across participating camps, they were not included as fixed effects in the model. Their potential influence is acknowledged, however. Thus, future studies should incorporate standardized indices of behavioural and spatial conditions to better distinguish pollution-related effects from concurrent stressors. Sample integrity may have been affected by pre-analytical factors such as the time interval between defecation and freezing. Although all faecal samples were placed in a cooled, insulated container and frozen within approximately 3–4 hours, the potential for partial degradation cannot be excluded. The exact time-to-freezing was not recorded per sample and therefore could not be included as a covariate in the statistical models. However, the sampling workflow was standardized across all camps to minimize inter-site variation. Faecal glucocorticoids have been reported to remain stable up to 48 hours under moderate temperatures (Larm et al., 2021), although reductions have been observed after 24 hours even at refrigeration conditions (Carbillet et al., 2023). However, the several freeze–thaw procedures could affect the faecal glucocorticoids concentrations, which should be avoided. The stability of oxidative damage markers such as 8-OHdG and MDA in faecal matrices has not been adequately characterized. However, similar time frames have been used successfully in comparable studies of Asian and African elephants under field conditions (Kosaruk et al., 2023b, 2023a; Oduor et al., 2024; Perera et al., 2025).

PM_2.5_ exposure data were derived from a central monitoring station located in Chiang Mai city, which may not fully capture local-scale variation at individual camps. However, prior studies have demonstrated that during high-pollution periods in Northern Thailand, regional biomass burning and atmospheric stagnation lead to highly correlated PM_2.5_ levels across urban and rural stations within the same air basin (Amnuaylojaroen et al., 2023; Jainontee et al., 2023). International literature similarly supports the use of a single fixed-site monitor to estimate PM_2.5_ exposure across geographically proximate areas under uniform pollution events (Yao et al., 2015; Shi et al., 2018; Yatkin et al., 2020). Nonetheless, local-scale differences in pollution levels remain a potential source of exposure misclassification.

## Conclusion

This study provided a rare longitudinal monitoring of multiple biomarkers to capture the effects of ambient PM_2.5_ on captive Asian elephants across tourist venues near Chiang Mai, Thailand, a region known for its annual pollution episodes. By combining repeated measures of oxidative DNA damage, lipid peroxidation and glucocorticoid metabolites, our approach offers a robust glimpse into how elephants physiologically respond to fluctuating air quality under real management conditions. However, several limitations are acknowledged. The observational nature of our design inherently precludes definitive conclusions about causality. Northern Thailand’s seasonal pollution coincides with prolonged dry periods and high ambient temperatures (Sirithian and Thanatrakolsri, 2022). This overlap makes it difficult to fully disentangle pollutant-specific effects from background climatic stressors, despite our models controlling for some environmental covariates. The moderate sample size, though sufficient to detect main patterns, may have limited our ability to resolve subtler interactions. In addition, feeding regimes and daily workloads vary between camps and seasons; some individuals may receive different quantities or types of supplements (e.g. bananas or sugar cane), while others participate more intensively in tourist activities. Such unmeasured nutritional and behavioural factors could influence oxidative or endocrine physiology independently of pollution exposure (Kosaruk et al., 2023a). While our systemic biomarkers reflect whole-body oxidative and stress status, they may not detect localized impacts such as direct lung tissue inflammation, which remains unmeasured in this context. Furthermore, our reliance on regional ambient PM_2.5_ data assumes uniform exposure across sites, yet elephants’ real inhalation dose likely varies with microclimate, shelter design and daily movement patterns. Direct measurements, using individual or enclosure-level samplers, would strengthen future estimates of true exposure.

Taken together, our multi-biomarker results provide insights into how elephants’ physiological responses fit within a broader mammalian context. The observed declines in oxidative markers during periods of intense pollution indicate that elephants may employ unique physiological or metabolic adaptations not typically observed in smaller mammals. The strong and consistent rise in fGCM with increasing PM_2.5_ aligns well with several studies in other species. Such alignment confirms that pollution acts as a genuine physiological stressor across taxa, even where oxidative marker patterns diverge. Compared with the seasonal elephant literature, which typically shows oxidative markers peaking in hot dry months (Kosaruk et al., 2023a), our observation that these markers dropped under extreme pollution suggests that episodic pollution imposes distinct pressures not fully explained by ambient heat alone. This divergence further emphasizes that targeted air pollution events may trigger physiological states not captured by general seasonality studies and that elephants, like other large mammals, can display both resilience and vulnerability through complex stress-response trade-offs. These findings highlight the need for incorporating air quality considerations into elephant welfare assessments and open new avenues for comparative studies on pollution resilience in elephants.

## Supplementary Material

Web_Material_coag008

## Data Availability

All biomarker data or analysed for this study are included in this article (and its supplementary information files). Daily PM_2.5_ data were obtained from a publicly available air quality database (Chiang Mai station), accessible via [World Air Quality Index project; https://aqicn.org/city/chiang-mai/]. Due to third-party data restrictions, raw PM_2.5_ values are not included in the Supplementary Materials, but the data are freely available from the original provider.
