# Supplementary material for: Ambient fine particulate matter exposure influences oxidative stress and glucocorticoid concentrations in captive Asian elephants in Thailand
Source: Conserv Physiol. 2026 Feb 17;14(1):coag008. doi: 10.1093/conphys/coag008 (PMC12910621; doi:10.1093/conphys/coag008)
Supplement: Web_Material_coag008 [file web_material_coag008.zip › Table S1.docx]

**Table S1** Comparison of models for each biomarker across different PM_2.5_ lag structures.

|  | **8-OHdG** | | **MDA** | | **fGCM** | |
| --- | --- | --- | --- | --- | --- | --- |
|  | **df** | **AIC** | **df** | **AIC** | **df** | **AIC** |
| **Lag 0** | 11 | 943.0256 | 11 | 278.8557 | 11 | 2064.528 |
| **Lag 1** | 11 | 943.0256 | 11 | 278.8557 | 11 | 2064.528 |
| **Lag 2** | 11 | 947.4178 | 11 | 278.5013 | 11 | 2062.212 |
| **Lag 3** | 11 | 946.0106 | 11 | 269.7038 | 11 | 2065.622 |

8-OHdG; 8-hydroxy-deoxyguanosine, MDA; malondialdehyde, fGCM; fecal glucocorticoid metabolites; AIC; Akaike’s Information Criterion
