# Supplementary material for: Ambient fine particulate matter exposure influences oxidative stress and glucocorticoid concentrations in captive Asian elephants in Thailand
Source: Conserv Physiol. 2026 Feb 17;14(1):coag008. doi: 10.1093/conphys/coag008 (PMC12910621; doi:10.1093/conphys/coag008)
Supplement: Web_Material_coag008 [file web_material_coag008.zip › Table S2.docx]

**Table S2.** Marginal and conditional R² values for the final linear mixed-effects models predicting three biomarker outcomes (8-OHdG, MDA, and fGCM).

| **Biomarker** | **Best Lag** | **Marginal R^2^** | **Conditional R^2^** | **Notes** |
| --- | --- | --- | --- | --- |
| 8-OHdG | Lag 0 | 0.259 | NA^†^ | Model singular; random effect variance ≈ 0 |
| MDA | Lag 3 | 0.159 | 0.389 |  |
| fGCM | Lag 2 | 0.080 | 0.249 |  |

8-OHdG: 8-hydroxy-2′-deoxyguanosine, MDA: malondialdehyde, fGCM: fecal glucocorticoid metabolites

*^†^ Conditional R² could not be estimated for the 8-OHdG model due to a boundary (singularity) issue, where the random-effect variance was approximately zero.*
